# Flaming Plasma Cell Leukemia

**DOI:** 10.4274/tjh.2016.0388

**Published:** 2018-05-25

**Authors:** Reza Ranjbaran, Habibollah Golafshan

**Affiliations:** 1 Shiraz University of Medical Sciences, School of Paramedical Sciences, Diagnostic Laboratory Sciences and Technology Research Center, Shiraz, Iran

**Keywords:** Plasma cell leukemia, Flame cell, Immunoglobin A

A 58-year-old man presented with anemia and splenomegaly. Peripheral blood smear indicated rouleaux formation along with 28% mononuclear cells with reddish-purple peripheral cytoplasm suspicious for plasma cells (PCs). Flow cytometric immunophenotyping of the peripheral blood revealed a large mononuclear population positive for CD38, CD138, and CD20 and negative for CD45, CD19, and CD56. Intracytoplasmic staining of kappa and lambda light-chains demonstrated lambda restriction. The serum protein electrophoresis pattern illustrated normal density in the γ-globulin region but an increase of about threefold in the β-globulin fraction.

Regarding these findings, the patient was more likely to be diagnosed with IgA monoclonal gammopathy [1]. However, a definitive diagnosis was made by immunonephelometric evaluation of serum immunoglobulins.This assay revealed 810 mg/dL IgG, 1595 mg/dL IgA, and 22 mg/dL IgM with a free kappa/lambda ratio of 0.14.

PCs have particular morphological features including oval-shaped structure and eccentric nuclei. IgA-secreting PCs have cytoplasm with a pinkish tinge associated with the presence of abundant glycoprotein and ribosomes and are totally known as flame cells (Figure 1). PC leukemia, in particular the IgA variant, is a rare and aggressive type of PC dyscrasia [2]. A well-prepared peripheral blood smear can be very helpful in diagnosing and determining the next diagnostic approach.

## Figures and Tables

**Figure 1:**
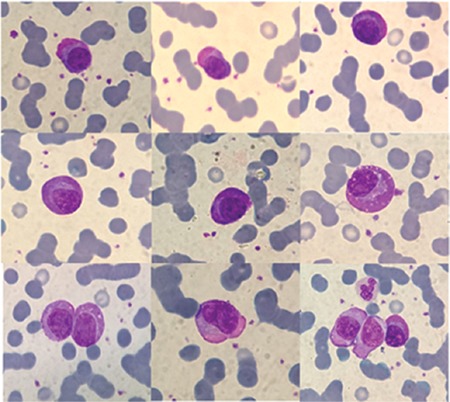
Flame cells
